# Genome-Wide Analysis and Expression Profiling of the Phenylalanine Ammonia-Lyase Gene Family in *Solanum tuberosum*

**DOI:** 10.3390/ijms23126833

**Published:** 2022-06-20

**Authors:** Fangyu Mo, Long Li, Chao Zhang, Chenghui Yang, Gong Chen, Yang Niu, Jiaxin Si, Tong Liu, Xinxin Sun, Shenglan Wang, Dongdong Wang, Qin Chen, Yue Chen

**Affiliations:** 1 State Key Laboratory of Crop Stress Biology for Arid Areas, College of Agronomy, Northwest A&F University, Yangling 712100, China; 2020055057@nwafu.edu.cn (F.M.); 2019055061@nwafu.edu.cn (L.L.); zhangchao520@nwafu.edu.cn (C.Z.); 2018060035@nwafu.edu.cn (C.Y.); 2020050129@nwafu.edu.cn (G.C.); 2019050076@nwafu.edu.cn (Y.N.); 2019055039@nwafu.edu.cn (J.S.); 2020055066@nwafu.edu.cn (T.L.); 2021055021@nwafu.edu.cn (X.S.); 2021055031@nwafu.edu.cn (S.W.); dongdong-1025@hotmail.com (D.W.); 2 College of Food Science and Engineering, Northwest A&F University, Yangling 712100, China

**Keywords:** potato, phenylalanine ammonia-lyase (PAL), evolution, expression, gene family

## Abstract

Phenylalanine ammonia-lyase is one of the most widely studied enzymes in the plant kingdom. It is a crucial pathway from primary metabolism to significant secondary phenylpropanoid metabolism in plants, and plays an essential role in plant growth, development, and stress defense. Although PAL has been studied in many actual plants, only one report has been reported on potato, one of the five primary staple foods in the world. In this study, 14 StPAL genes were identified in potato for the first time using a genome-wide bioinformatics analysis, and the expression patterns of these genes were further investigated using qRT-PCR. The results showed that the expressions of StPAL1, StPAL6, StPAL8, StPAL12, and StPAL13 were significantly up-regulated under drought and high temperature stress, indicating that they may be involved in the stress defense of potato against high temperature and drought. The expressions of StPAL1, StPAL2, and StPAL6 were significantly up-regulated after MeJa hormone treatment, indicating that these genes are involved in potato chemical defense mechanisms. These three stresses significantly inhibited the expression of StPAL7, StPAL10, and StPAL11, again proving that PAL is a multifunctional gene family, which may give plants resistance to multiple and different stresses. In the future, people may improve critical agronomic traits of crops by introducing other PAL genes. This study aims to deepen the understanding of the versatility of the PAL gene family and provide a valuable reference for further genetic improvement of the potato.

## 1. Introduction

Phenylalanine ammonia-lyase (PAL; EC 4.3.1.5) catalyzes the deamination of L-phenylalanine to trans-cinnamic acid, which catalyzes the first step in the phenylpropanoid pathway and is conserved in virtually all eukaryotes [1]. This pathway engenders many aromatic metabolites, such as flavonoids, isoflavonoids, and lignins [2,3]. Therefore, PAL is involved in the biosynthesis of a wide range of secondary metabolites. These metabolites are critically important for the growth and development of plants. In addition to its essential role in plant growth and development, PAL is also a key enzyme in plant stress responses. Its expression activity is affected by drought [4], pathogen attack, tissue wounding, extreme temperatures, UV irradiation, deficiency of nutrition [5], and exposure to plant signaling molecules, including jasmonic acid (JA) [6], SA [7], and abscisic acid (ABA) [8]. After being stimulated by multiple stresses, expression of the PAL gene rapidly induced PAL gene expression at the transcriptional level [9]. This indicates that the PAL gene plays a crucial role in plants resisting various biotic and abiotic stresses. Therefore, it will become possible to improve plants’ resistance to multiple stresses by increasing the expression of the PAL gene.

The PAL gene was first isolated from Hordeum vulgare [10] and has since been found across various organisms, including plants, viruses, algae, and fungi [11]. This shows that PAL widely exists in different creatures on earth. It is usually encoded by small multi-gene families [12]; the PAL gene family often contains multiple family members, the number of which varies from species to species. For instance, the number of PAL gene copies is 4 in *Arabidopsis thaliana* [13] and *Nicotiana tabacum* L. [14], 5 in *Populus trichocarpa* [15], 7 in *Medicago sativa* [16] and *Cucumis sativus* L. [17], 9 in *Oryza sativa* L. [15], and 12 in *Citrullus lanatus* [18]. The expression of the PAL gene in plants is tissue-specific, its relative molecular mass weight is 220,000–340,000, and it is an oligomer composed of four 77,000 to 83,000 subunits [19]. In higher plants, PAL activity varies significantly with the stage of development, with cell and tissue differentiation, and upon exposure to various kinds of stress [20]. Take *Arabidopsis thaliana* as an example; the active PAL isoforms are encoded by four genes designated as AtPAL1-4 [13]. Expression studies of the AtPALs have shown that AtPAL1 expression is localized to vascular tissue, AtPAL2 and AtPAL4 are expressed in seeds [21], and AtPAL3 is expressed only at basal levels in stems [22]. Studies on stress induction of these four PALs found that only AtPAL1 and AtPAL2 were induced under low temperature and reduced nitrogen content [23]. These prove that there may be significant differences in the expression of the PAL gene in different plants, tissues, and stresses.

Potato (*Solanum tuberosum* L.) is one of the essential therophyte Solanaceae herbaceous species, and it is planted at >19 million hectare worldwide [24]. There are many studies on phenylalanine ammonia-lyase in potato; for example, rapid and local PAL gene activation has been demonstrated in fungus-infected potato leaves [25], expression during tissue differentiation [20], and the effects of injury stress on the activity of this enzyme in potato [26]. There are research studies showing that the levels of total phenolic and flavonoid compounds in potato are controlled primarily by PAL and CHS gene expression, and that the expression of PAL, CHS, and AN1 are regulated in a coordinated manner [27]. The PAL gene plays a key and decisive role in the metabolism of potato bioactive substances. In addition, research has found that biogenic elicitors (chitosan and its complex with salicylic acid) and an immunosuppressor (laminarin) were shown to increase the activity of L-phenylalanine ammonia-lyase and protein synthesis in potato tubers [28]. The results of this study suggest that potato may have the same expression specificity as other plants where PAL has been studied. So far, the potato has been found to contain only about 40–50 phenylalanine ammonia-lyase (PAL) genes/haploid genome and at least 10 active genes [25]. The existing literature reveals only a very general understanding of the potato PAL gene family members, and no one has conducted in-depth and detailed research on the topic.

We identified this study’s potato PAL gene family and obtained 14 potato PAL genes. The physical and chemical properties, sequence characteristics, phylogenetic evolution, chromosome location, tandem replicated genes, gene structure, conservative motif, cis-regulatory element prediction, protein–protein interaction networks, interspecies collinearity, gene expression, and qRT-PCR analysis of potato StPAL protein were studied using bioinformatics analysis, which provided some clues for further revealing the function of the potato PAL gene family.

## 2. Results

### 2.1. Identification of Members of PAL Gene Family in Potato

Using the *Arabidopsis* PAL gene family protein sequences, the potato genome database was searched using the NCBI “blastp” software program, and 20 potato protein sequences were screened. Simultaneously, the sample was screened using HMMER software according to the PAL gene domain (PF00221). Two repeated sequences were found in the screening results and could be deleted. After sequence alignment, it was found that Soltu.dm.03G004920 (1), Soltu.dm.03G011450 (2), Soltu.dm.05G017030 (3), and Soltu.dm.s001510 (4) did not contain the AlA-SER-Gly A highly conserved MIO (4-methylidene-imidazole-5-one) electrophilic group. Although (1) and (3) do not contain the MIO domain, their sequences are complete and valuable for research and can be retained, while (2) and (4) do not contain the MIO domain, and their sequences are incomplete. Genes can be deleted. In addition, we removed two genes containing the MIO domain but with more than 50% sequence defects, and, finally, we identified 14 PAL genes from the potato genome (Table 1). To verify the correctness of the preliminary identification results, we used SPDE2.0 software for further comparison and screening to confirm that the selected genes were the same.

Detailed information regarding the 14 PAL genes, including gene names, gene IDs, chromosome locations, open reading frame (ORF) lengths, exon numbers, basic protein parameters, and subcellular localization predictions, are provided in the Table 1. The ORF size of the StPAL protein ranged from 1956–2172 bp. The length of the protein is 651–723 amino acids, the molecular weight is 70.75–78.82 kDa, and the predicted pI value is 5.41–7.97.

### 2.2. Phylogenetic Analysis of PAL Gene Family in Potato

**Figure 1:**
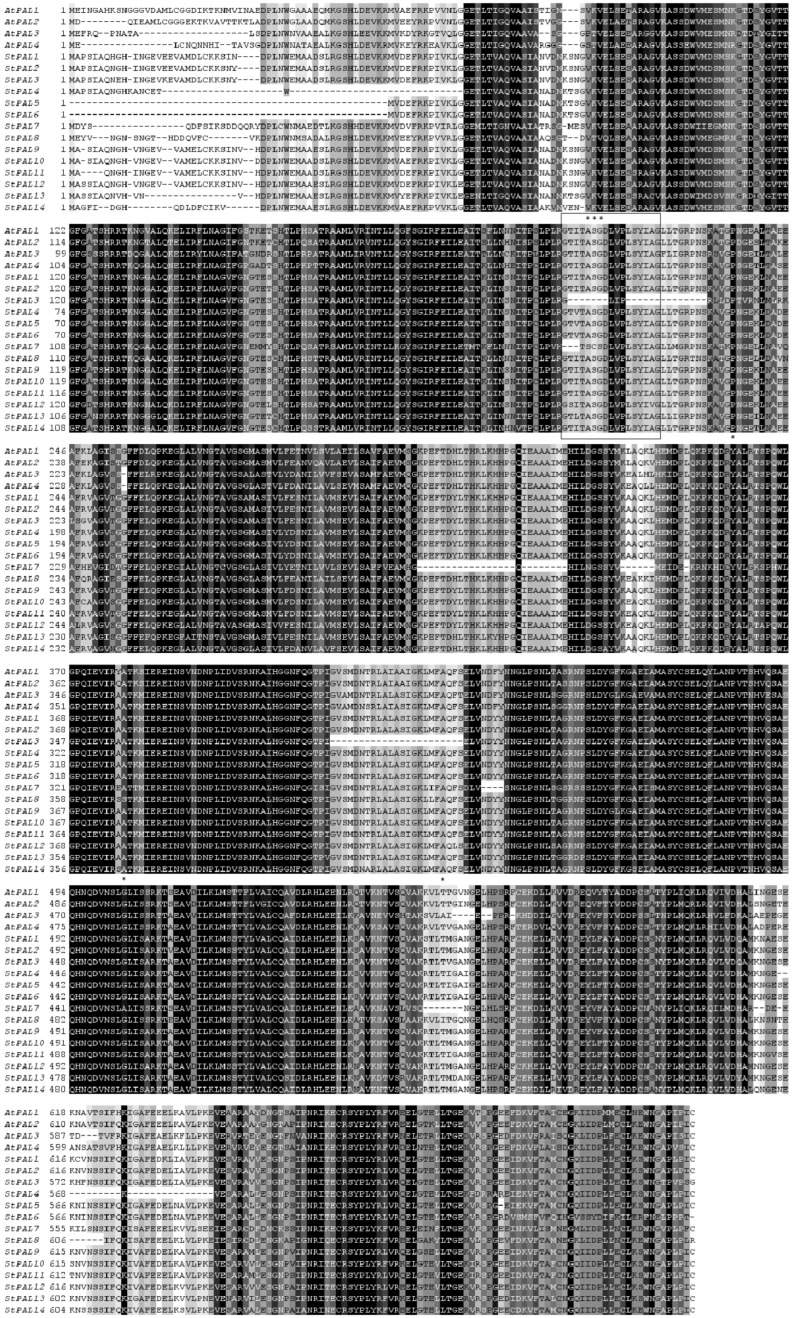
Multiple sequence alignment of the fourteen potato PAL protein sequences and *Arabidopsis* PAL proteins. The alignment was performed using MAFFT with defaults, followed by shading with conservation. The darker the color of the region, the more conservative it is. A box circles the active sites of PAL. The conserved enzymatic active site Ala-Ser-Gly is marked with black asterisks (***). The gaps are indicated as dashes.

In *Arabidopsis*, multiple sequences were compared between StPAL and AtPAL proteins (Figure 1). All PAL proteins were highly conserved. Consistent with other plant PALs, the StPAL protein has four functional domains. Taking StPAL1 as an example, it includes an N-terminal domain (1–29), an MIO domain (30–268), a core domain (269–534 and 652–723), and an inserted shielding domain (535–651) [9]. Similar to PAL contrast among other plants, the most significant sequence divergence occurred in the N-terminal region (Figure 1), such as in soybean [29], raspberry [30], and watermelon [18]. Except for StPAL3 and StPAL7, the active site GTITASGDLV(I)PLSYIAG of PAL was detected in all other PAL genes, which contained a highly conserved MIO electrophilic group composed of Ala-Ser-Gly. These proteins also contain two conserved residues in the core domain (Tyr358 and Gly501, numbered according to AtPAL1) that have been reported to be critical for PAL enzymatic activity [9]. However, among the fourteen PAL genes, only StPAL7 was abnormal, and nonpolar Ala residues replaced its polar Gly residues. In the shielding domain, a post-translational phosphorylation site (Thr556, numbered according to AtPAL1), which researchers detected in the bean [29], cucumber [17], and alfalfa [16] PAL sequences, was also present in most of the StPAL proteins. The exceptions occurred in one AtPAL (AtPAL3) and two StPALs (StPAL7 and StPAL8). In AtPAL, the polar Thr residue is replaced by the nonpolar residue Ala, and in StPAL8, Thr is replaced by Ile. Neither Val nor Ala could be phosphorylated. No amino acids were detected in StPAL7, and neither StPAL3 nor StPAL7 has the MIO domain. Therefore, it is speculated that these two genes may have different physiological functions and may be redundant or unused PAL.

**Figure 2:**
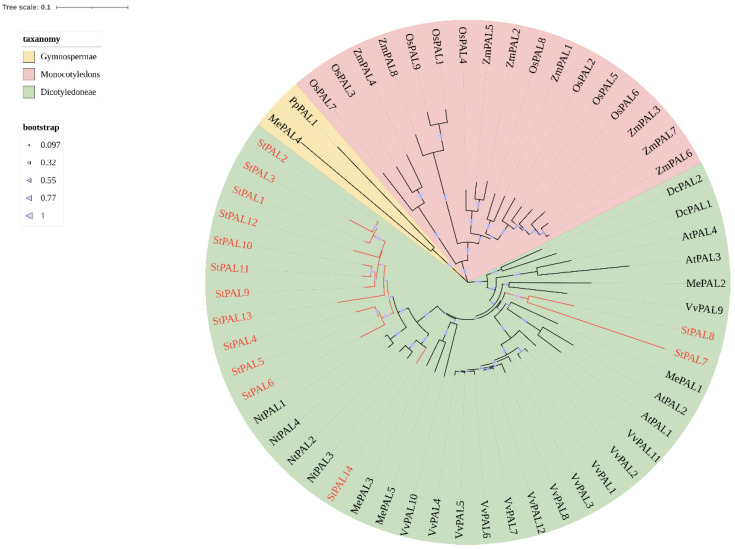
Phylogenetic analyses of the plant PAL proteins. The conserved PAL proteins from potato, rice, and *Arabidopsis* were aligned using the ClustalW function of MEGA11, and the phylogenic tree was constructed using the NJ method with bootstrapping analysis (1000 replicates).

To evaluate the evolutionary relationship between potato PAL proteins, we constructed a neighbor-joining tree (NJ) with MEGA 11.0 software and performed a phylogenetic analysis. In addition to the PAL proteins of potato, the phylogenetic tree includes PAL proteins of model dicots (*Arabidopsis thaliana*, *Nicotiana tabacum* L., *Vitis vinifera* L., and *Manihot esculenta Crantz*) and monocots (*Oryza sativa* L., *Zea mays* L., and *Dioscorea oppositifolia* L.), as well as PALs of gymnosperm (*Pinus pinaster*) protein (Table A1). As shown in Figure 2, the tree was divided into the following three groups: dicots, monocots, and gymnosperms. As expected, potato PALs were grouped into dicotyledonous groups, with nine PALs forming a potato subgroup. Three StPALs genes (StPAL14, StPAL8, StPAL7) belong to dicotyledonous plants but are not in the potato subgroup. Among them, StPAL8 and StPAL7 clustered with *Arabidopsis* and grape PALs, indicating that StPAL8 and StPAL7 may perform different functions from other PAL genes in potato. Among all tuber PALs, some potato PALs aggregated with each other into a triple. In total, we found three such potato PAL triples. At the same time, it was also found that all potato PALs clustered in the same branch with tobacco, indicating that the relationship between potato and tobacco PAL gene family is relatively close.

### 2.3. Chromosomal Location and Tandem Duplication Genes of Potato PAL Gene Family

**Figure 3:**
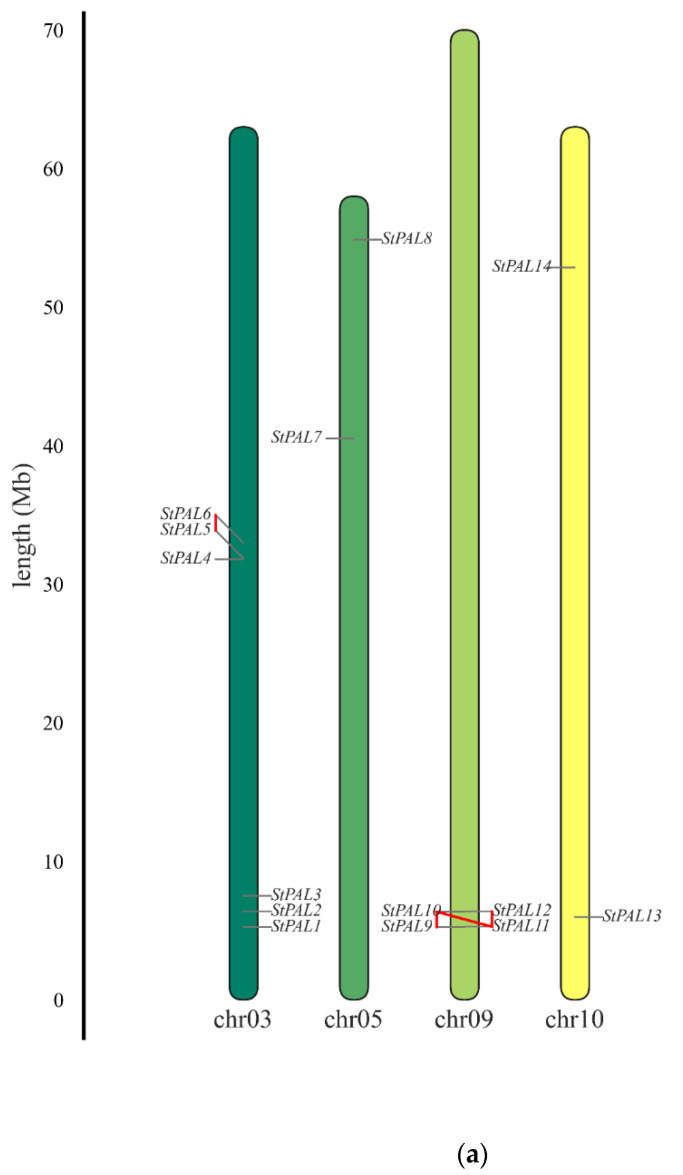

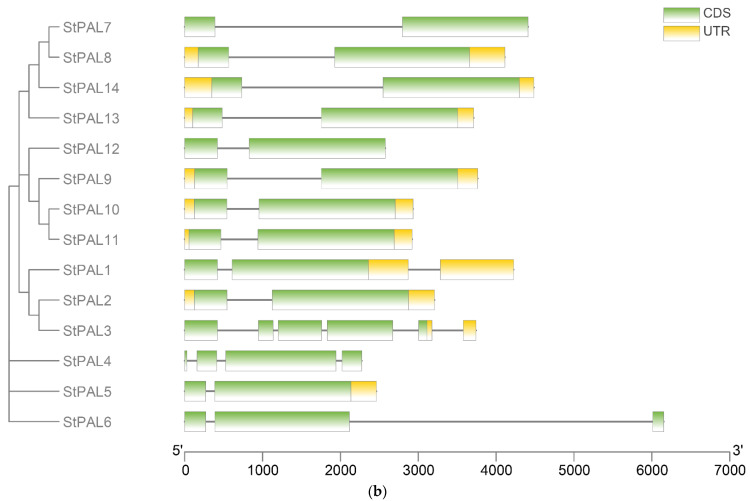
Chromosomal location and gene structure for PAL proteins. (**a**) Chromosomal distribution of potato PAL genes. The red box indicates tandem duplicate genes. (**b**) Green color bars represent the exon, lines represent the intron, and yellow color bars indicate the untranslated region (UTR) both at 50 and 30.

Chromosomal mapping results showed that 14 potato PAL genes were randomly and unevenly distributed on chromosomes 3, 5, 9, and 10. There are six PAL genes on chromosome 3, four PAL genes on chromosome 9, and two PAL genes on chromosomes 5 and 10, respectively (Figure 3a). There are various mechanisms for gene family amplification, including polyploidy, fragment duplication, tandem duplication, transposable elements, etc. [31]. To study the genome duplication event of the potato PAL gene, we identified two tandem repeats (STPAL5/6) on chromosome 3 and three tandem repeats (StPAL9 and StPAL10, StPAL10 and StPAL11, StPAL11 and StPAL12) on chromosome 9, according to defined criteria.

From the perspective of the StPAL gene structure, the number of exons of StPAL gene family members is between two and five. Indeed, StPAL3, StPAL4, and StPAL6 contain five, four, and three exons, respectively, while other PAL gene family members have only two. Compared with exons, introns of StPAL gene family members were more stable. The StPAL4, StPAL6, StPAL7, and StPAL12 do not contain introns, StPAL5 has only one intron, and other members of this family all contain two introns (Figure 3b). Therefore, the structural differences among members of the StPAL gene family are insignificant, and only a few members have differentiated, indicating that the original structure of the StPAL gene is not complicated. The complexity is the gene after mutation evolution, so the PAL gene’s evolution process needs more meticulous research.

### 2.4. Cis-Acting Elements Analysis and Conserved Motif Identification

**Figure 4:**
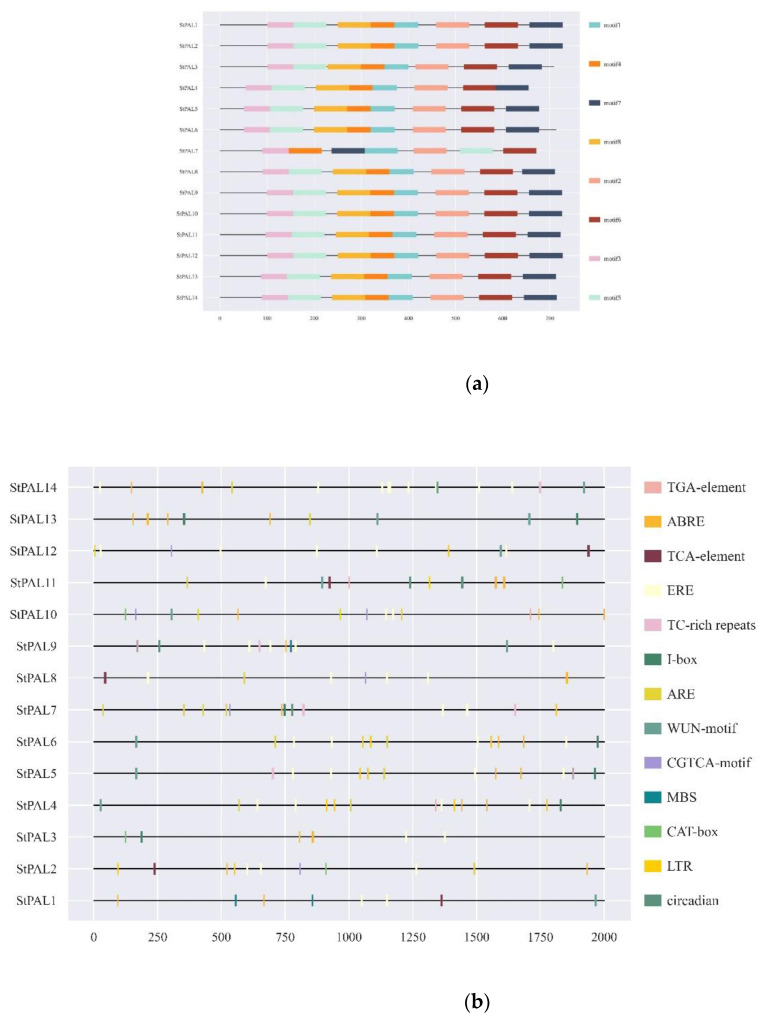
Conserved motif identification and cis-acting elements analysis for PAL proteins. (**a**) Different colors indicate different motifs. (**b**) Cis-acting element of 1500 bp sequences upstream of the potato StPAL gene. This study used the PlantCARE database to predict the elements.

To further study the conservation of potato PAL protein sequences and the difference in motif composition among potato proteins, the conserved motifs of potato PAL protein sequences were further analyzed using MEME. The results showed that the structures of the potato PAL family genes were not wholly consistent. Except for PAL7, other PALs contained eight conserved structures, while PAL7 had only seven conserved structures and did not contain Motif8. All conserved structures were 50 amino acids in length (Figure 4a, Table 2). According to the conserved structure and sequence alignment results, Motif1 contains a conserved residue (Tyr358) located in the core domain, and Motif7 contains another conserved residue (Gly501) situated in the core domain. These two residues are critical for PAL enzymatic activity [9,14]. Since PAL3 and PAL7 do not contain MIO conserved domains, none of the motifs have the MIO domain, so the program did not predict them. The protein numbers of the eight motifs are all PF00221. Except for Motif7, the other 7 are distributed in 14 StPAL proteins, indicating that the sequence of StPAL proteins is conserved.

To further study the regulation mechanism of the StPAL gene under abiotic stress, according to the different functions of different cis-acting elements, 13 cis-acting elements related to growth and development, hormone, and stress response were outlined. The promoter of the StPAL gene contains many homeopathic regulatory elements. The most StPAL4 has 15 cis-acting elements, and the least StPAL3 has 6 cis-acting elements. There are five stress response elements, as follows: anaerobic induction (ARE), light response (I-box), low temperature response (LTR), drought-responsive (MBS), mechanical damage (WUN-motif), and six hormone response elements, namely stress reaction (TC-rich repeats), abscisic acid (ABRE), methyl jasmonate (CGTCA-motif), salicylic acid (TCA-element), auxin (TGA-element), and ethylene (ERE). The two growth and developmental response elements are meristem expression regulation (CAT-box) and circadian rhythm regulation (Figure 4b). Among the StPAL genes, abscisic acid (ABRE), ethylene (ERE), and anaerobic induction (ARE) have the most significant number of three action elements, as they have 33, 49, and 19, respectively. Except for StPAL12, these three elements are randomly distributed among other genes, indicating that these genes are involved in plant responses to oxygen and hormones, such as abscisic acid and ethylene. Cis-element analysis showed that the StPAL gene was closely related to plants’ abiotic stress, growth, and hormone secretion.

### 2.5. Potato PAL Protein Interaction Analysis

**Figure 5:**
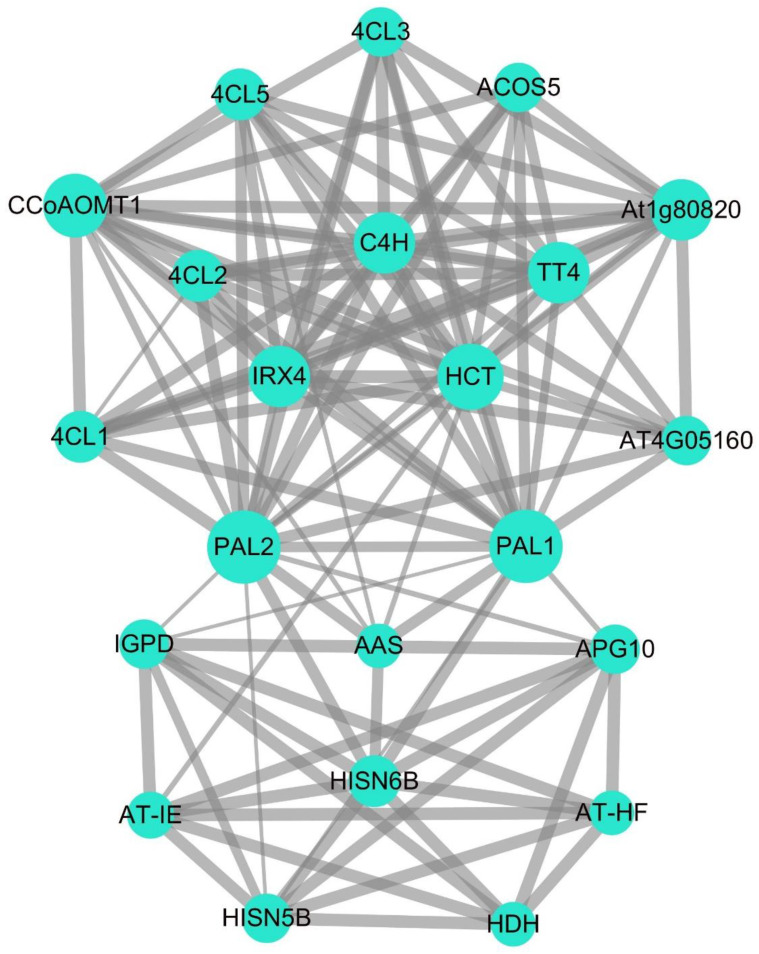
Function interaction network of StPAL proteins. The size of the circle indicates how often the protein appears (the larger the area, the higher the frequency). The thickness of the line segment between proteins indicates the combined score of correlation degree between two proteins (the thicker the line segment, the higher the score).

Figure 5 shows a partially predicted PAL gene regulatory network and protein– protein interaction network of potato. Only one subnet was identified using the STRING database to predict the interconnected genes in the network map of potato PAL protein functional relationships and (species model selection *Arabidopsis thaliana*) protein interactions. The top three associations with PAL1 and PAL2 are Cinnamate 4-hydroxylase (C4H), 4-Coumarate: Coenzyme A Ligase (4CL1, 4CL2, 4CL3), and HISN6B. The C4H and 4CL (1, 2, 3) are the key enzymes in the plant phenylpropane synthesis pathway. The protein activity and transcriptional abundance of CH4 directly affect plants’ biosynthesis of flavonoids and aromatic compounds [32]. On the other hand, 4CL can catalyze the formation of cinnamoyl-CoA from 4-coumaric acid, which plays a vital role in the regulation of phenylpropanoid metabolic pathways, such as flavonoids, lignin, coumarin, sporopollenin, and chlorogenic acid [3]. The study shows that the potato PAL gene family is also involved in the phenylpropane metabolism pathway and may be the key enzyme in the synthesis of flavonoids. The role of HISN6B is to encode a protein that, to a certain extent, compensates for the loss of HISN6A (AT5G10330) and functions as a histidine-phosphate transaminase in histidine biosynthesis. Therefore, PAL may also be involved in the synthesis of phosphate compounds.

### 2.6. Collinear Analysis of PAL Genes in Potato and Arabidopsis

**Figure 6:**
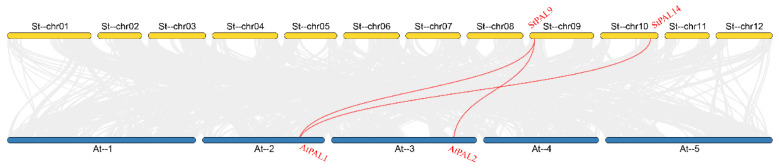
Homology analysis of PAL genes between potato and *Arabidopsis*. Gray lines represent collinear blocks between potato and *Arabidopsis* genomes. Red lines represent syntenic PAL gene pairs between potato and *Arabidopsis*.

The gene collinearity study showed that potato and *Arabidopsis* shared three pairs of homologous PAL genes, including two potato PAL genes and two *Arabidopsis* PAL genes (Figure 6), indicating that the potato and *Arabidopsis* PAL genes families have some kind of homologous evolutionary relationship. Among them, StPAL9 is collinear with at least two AtPAL genes, and one of the two *Arabidopsis* genes collinear with it is collinear with StPAL10, indicating that these four genes may have similar functions in potato and *Arabidopsis*, and that they play an important role in the evolution of the PAL gene family. The results showed that there was no collinear relationship between StPAL1, StPAL2, StPAL3, StPAL4, StPAL5, StPAL6, StPAL7, StPAL8, StPAL11, StPAL12, StPAL13, StPAL14, and AtPAL genes, indicating that these genes may be specific genes in potato evolution.

### 2.7. Tissue Expression of Potato PAL Gene and Expression Analysis of Stress Treatment

**Figure 7:**
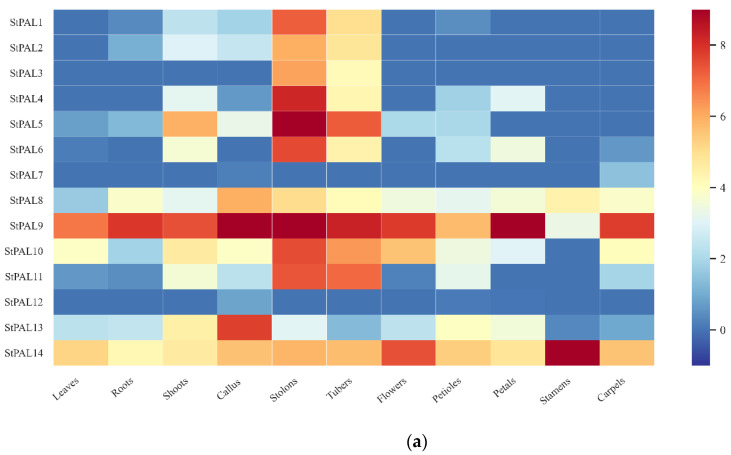

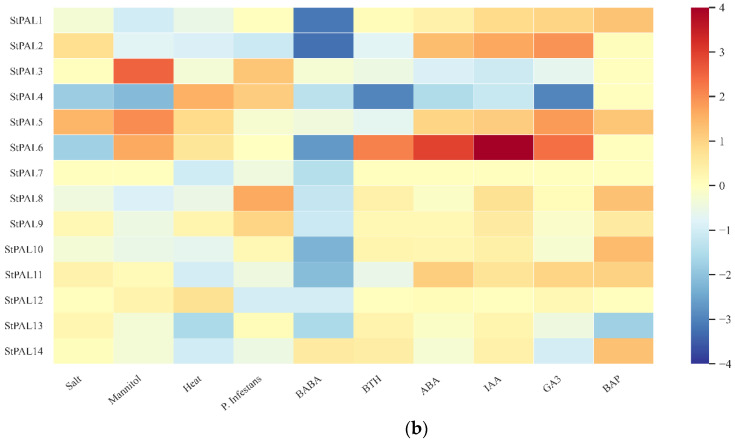
(**a**) Heatmap shows the expression of the StPAL gene in 12 tissues, including the leaves, roots, shoots, callus, stolons, tubers, flowers, petioles, petals, stamens, and carpels. Red indicates high relative gene expression, whereas blue indicates low relative gene expression. (**b**) Heatmap of the expression profile of potato PAL genes under 10 different biotic or abiotic stresses. Abiotic stresses included salt, mannitol, and heat; biotic stresses included P. infestans, β-aminobutyric acid (BABA), benzothiadiazole (BTH), and P. infestans; other stress responses were mainly induced by the following four plant hormones: 6-benzylaminopurine (BAP), auxin (IAA), abscisic acid (ABA), and gibberellin glutathione (GA3). Red indicates gene upregulation, while blue indicates gene downregulation.

To study the role of potato PAL genes in growth and development, we obtained transcriptome data from the potato genome database, found RNA-seq data of PAL genes in different tissues and under various stresses, and drew heatmaps (Table A2 and Table A3). We studied the expression levels of StPAL genes in leaves, roots, shoots, callus, stolons, tubers, flowers, petioles, petals, stamens, carpels, and other tissues (Figure 7a), as well as the expression levels under stresses, such as salt, mannitol, heat, P. infestans, β-aminobutyric acid (BABA), benzothiadiazole (BTH), abscisic acid (ABA), auxin (IAA), gibberellin glutathione (GA3) and 6-benzylaminopurine (BAP) (Figure 7b).

The PAL gene was detected in all tissues, and StPAL8, StPAL9, StPAL13, and StPAL14 were expressed to a high degree in all tested tissues. Among them, the expression level of StPAL9 in stolon reached an overall peak. It indicated that these four genes have a tremendous regulatory role in potato growth and development. However, StPAL7 and StPAL12 were mainly expressed in carpels and callus. Nonetheless, the expression levels were still low, indicating that these genes may not play a significant role in regulating plant development. The StPAL3 was only expressed in stolons and tubers, and the expression level was moderate. In addition, we found that, except for StPAL7 and StPAL12, the overall expression of other PALs genes was concentrated in stolons and tubers, indicating that PAL genes are closely related to the growth and development of stolons and tubers. Then, we counted the expression of StPAL genes under 10 kinds of stresses. We found that 13 genes were down-regulated under β-aminobutyric acid stress, and that StPAL6 was more sensitive to various pressures. The StPAL genes mainly responded to salt, abscisic acid, auxin, gibberellin, and 6-benzylaminopurine treatment, expressing that five, five, seven, five, and seven genes were significantly up-regulated, respectively.

### 2.8. Expression Analysis of StPAL Genes in Different Treatments

**Figure 8:**
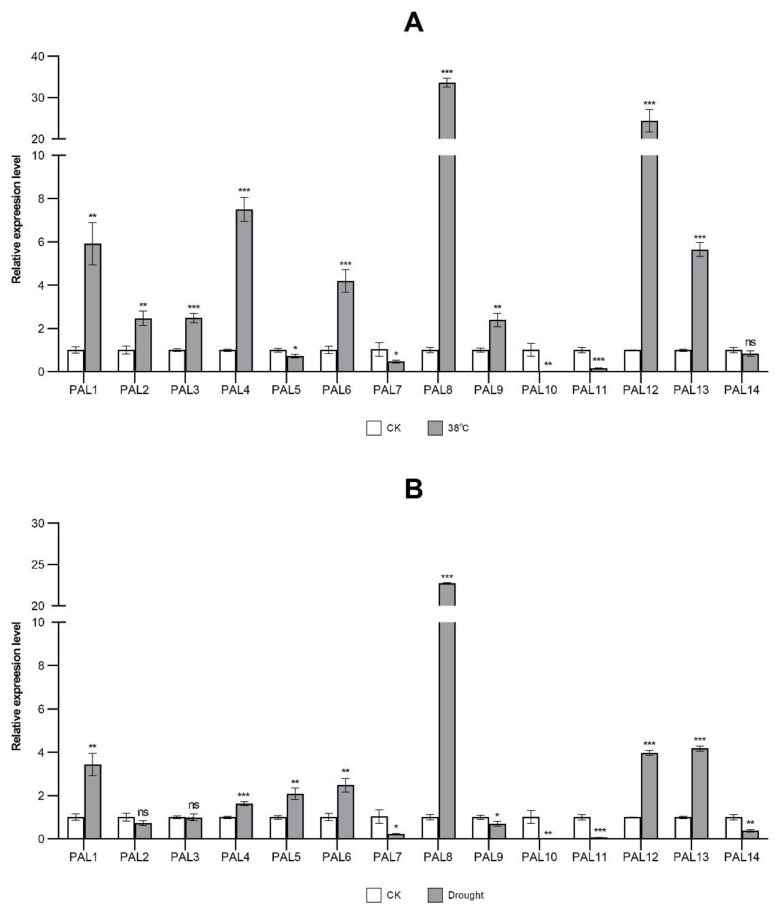

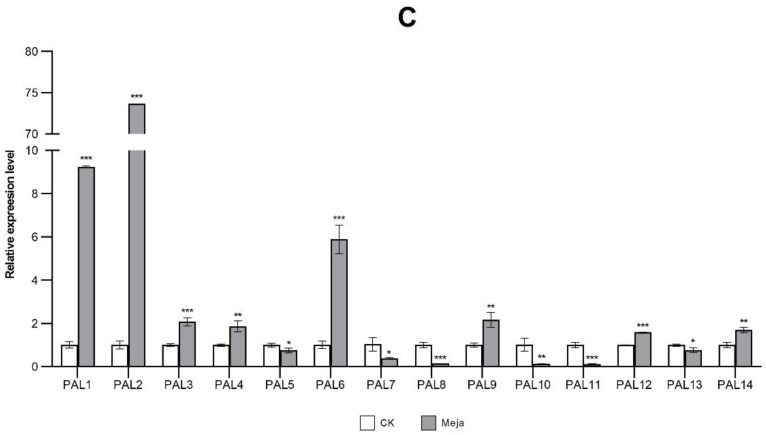
Differential expression of the StPAL gene family in response to abiotic stress and hormone induction. (**A**) Relative expression level at 38 °C. (**B**) Relative expression level under drought treatment. (**C**) Relative expression level under MeJa treatment. (* *t*-test *p*-value < 0.05, ** *t*-test *p*-value < 0.01, *** *t*-test *p*-value < 0.001).

The presence of many environmental signal-responsive cis-elements in the promoters of the StPAL genes (Table 2) suggests that the expression of StPALs might respond to various abiotic stresses. To test this hypothesis, we conducted qRT-PCR analyses to quantify the expression levels of the StPAL genes in response to stress treatments of potato roots (Table A4). Root tissue was selected because the expression of all of the StPALs could be detected. Potato Desiree materials were treated with high temperature, drought, and methyl jasmonate (MeJA). Then, the expression of the StPAL gene was detected by real-time quantitative qRT-PCR to analyze the expression of 14 StPAL genes under high temperature, drought, and MeJA stress (Figure 8). The results showed that all three stresses altered the expression levels of PALs in all potatoes, but the extent of the changes varied by gene and stress.

As shown in Figure 8A, potato seedlings under high temperature stress (38 °C) showed an up-regulation of nine StPAL genes. The qRT-PCR results in the StPAL7 and StPAL11 were consistent with the results in the RNA-seq data. The StPAL1, StPAL2, StPAL3, StPAL4, StPAL6, StPAL8, StPAL9, StPAL12, and StPAL13 were significantly upregulated, and StPAL7, StPAL10, and StPAL11 were significantly downregulated, especially StPAL8 and StPAL12, the expression of which increased 33.70- and 24.47-fold after 6 hours of treatment, respectively. The StPAL1, StPAL4, StPAL6, and StPAL13 increased 5.92-, 7.51-, 4.21-, and 5.66-fold, respectively. The remaining three StPALs (StPAL2, StPAL3, StPAL9) showed a slight increase, between 2.40- and 2.48-fold. The expression levels of PAL7 and PAL11 were significantly decreased by 0.47-and 0.17-fold, respectively. The StPAL10 has minimal expression. The expression levels of StPAL5 and StPAL14 did not change much.

Drought is another common stress that plants have to cope with. Under this stress, StPAL1, StPAL5, StPAL6, StPAL8, StPAL12, and StPAL13 were significantly upregulated, and StPAL7, StPAL10, StPAL11, and StPAL14 were significantly downregulated. The qRT-PCR results in the StPAL1 were consistent with the results in the RNA-seq data. This was especially true of StPAL8, as its expression increased 22.73-fold after 6 hours of treatment. The expression of StPAL1, StPAL5, StPAL6, StPAL12, and StPAL13 increased 3.45-, 2.09-, 2.42-, 3.98-, and 4.18-fold, respectively. The expression levels of PAL7 and PAL14 were decreased 0.23- and 0.38-fold, respectively. The StPAL10 and PAL11 have minimal expression. The expression levels of StPAL2, StPAL3, StPAL4, and StPAL9 did not change much, showing a slight change of between 0.70- and 1.63-fold (Figure 8B).

Under MeJA treatment, StPAL1, StPAL2, StPAL3, StPAL4, StPAL6, and StPAL9 were significantly upregulated, while StPAL7, StPAL8, StPAL10, and StPAL11 were significantly downregulated. The qRT-PCR results in the StPAL2 and StPAL9 were consistent with the results in the RNA-seq data. Among them, StPAL2 had the highest expression, reaching 73.65-fold. The expression of StPAL1, StPAL3, StPAL6, and StPAL9 increased 9.23-, 2.08-, 5.89-, and 2.16-fold, respectively. The StPAL4, StPAL5, StPAL12, StPAL13, and StPAL14 showed a slight change of between 0.75- and 1.87-fold. The expression levels of PAL7, PAL8, PAL10, and PAL11 were decreased by 0.39-, 0.14-, 0.12-, and 0.12-fold, respectively (Figure 8C).

## 3. Discussion

In this study, we identified 14 StPAL genes from the potato genome. Joos et al. studied the potato PAL gene in 1992 [25], but they did not explicitly analyze its gene family, so this is the first time that the PAL gene family has been described in potatoes. The PAL gene family exists universally in high plants. Joos et al. (1992) found that potatoes have at least 10 PAL genes, possibly many more. In this study, we identified 14 PAL genes in potatoes, consistent with previous predictions [25]. Furthermore, we found the core domain of StPAL genes to be highly conserved through conservative motif analysis and multisequence alignment (Figure 1 and Figure 4a). The results show that the genes we screened are correct, and all of them are valuable for research. Meanwhile, the PAL gene has a high degree of conservation in the process of evolution. Although StPAL3 does not have the MIO domain, it has been proved that the gene has similar biological functions to other StPALs after experimental verification, so it is still a member of the StPAL gene family.

In our study, 14 StPALs of the potato PAL gene family were arranged on chromosomes 3, 5, 9, and 10, respectively (Figure 3a), and the sequences of all genes were highly similar (Figure 1). This indicates the existence of one or more tandem repeats, so we analyzed the tandem repeats of the potato PAL gene during chromosomal localization. Two tandem repeats were found on two and four StPAL genes (Figure 3a). In addition, our analysis of the evolutionary tree of potato PAL protein showed that several pairs of StPALs with tandem repeats were clustered with most other StPAL proteins, and three StPALs were clustered with other plant PAL proteins (Figure 2). This result suggests that such duplication events occurred after the potato split from the other dicots. In the present study, there were two exons within most of the 14 PAL members (78.6% of cases). As can be seen from the clustering of Figure 3b, StPAL proteins with similar exon/intron structures are more likely to cluster together, which is consistent with the results of PAL gene family analysis in most plants, such as maize [33], pear [34] and *Juglans regia* [35]. Among them, the intron/exon organization of StPAL7-StPAL14 is very similar. These StPALs are clustered together, and all contain two exons and one intron. The intron/exon organization structure of StPAL1-StPAL6 shows diversity, with the number of introns ranging from two to six. These differences are caused by evolutionary continuities, which affect the number of introns. Such differences in gene structure are likely to lead to functional differences in genes. Asma Ayaz et al. (2021) observed a similar situation in the LACS gene family. However, the study included a much wider variety of plants, so this difference was more pronounced in the study [36].

Thanks to the development of high-throughput sequencing techniques, the function of PAL genes has now been identified in many kinds of plants. This is consistent with our protein interaction analysis results (Figure 5). There is a strong correlation between PAL protein and enzymes involved in the synthesis pathway of phenylpropane, flavonoid, and cinnamyl. The network interaction diagram also indicates that PAL may participate in synthesizing phosphate compounds. Still, the specific role of PAL in the reaction remains in need of further study. The PAL genes are widely distributed throughout the genome of most plants. For example, in *Arabidopsis thaliana*, AtPAL1, AtPAL2, AtPAL3, and PAL4 are located on chromosomes 2, 3, 5, and 3 respectively [37]. In tobacco, numerous duplicated MEMBERS of the PAL gene family do not cluster together [38]. Phylogenetic trees show that PAL genes can be divided into three distinct clades, as follows: monocotyledons, dicotyledons, and gymnosperms (Figure 2). This suggests that functional differentiation of PAL genes may have occurred when monocotyledons and dicotyledons separated (165 million years ago, in Myanmar), which is consistent with the results of *Medicago truncatula* PAL [39] and watermelon PAL [40]. In the dicot subgroup, the PAL genes from potato were most closely related to those of tobacco, indicating that the expansion of the StPAL gene family might have occurred before the speciation of tobacco and potato. Meanwhile, Figure 6 also showed that only StPAL9 and StPAL14 among the 14 StPAL genes had apparent homology with *Arabidopsis thaliana*. The copy number of PAL genes also varies from plant to plant, with most being between three and nine. For example, there are four members in *Arabidopsis thaliana* [13] and tobacco [14]. However, more than 20 members have been identified in tomato, although these are mostly inactive [38]. The same is true of the 14 potato PAL genes we studied. Indeed, StPAL7, StPAL10, StPAL11, and StPAL14 are relatively static (Figure 8). Different plants contain different numbers of PAL genes, and the size of the genome and gene duplication events cause these differences [41]. The LACS gene family also has other gene numbers in various plants [42]. Additionally, there are more tandemly duplicated genes in the StPALs, which confirms this.

The PAL mainly catalyzes phenyl propyl to produce trans-cinnamate esters, which is the first step of the whole reaction pathway, and the products are precursors of various secondary metabolites [43]. The silencing or loss of PAL can hinder the average growth and development of plants. For example, inhibition of PAL expression in tobacco can lead to stunted growth, changes in leaf structure, changes in petal morphology and pigment, and reduced pollen viability [44]. In *Arabidopsis thaliana*, changing the phenotype of PAL1/PAL2 double mutants leads to sterility, lignin reduction, and ultrastructural changes in secondary cell walls [21]. In addition, Pal quadruple knockout also resulted in developmental delay and sterility of mutants, and enhanced susceptibility to Pseudomonas syringae [39]. In potatoes, 11 StPAL genes were expressed in stolons and tubers but at low levels in other tissues (Figure 7a), suggesting a redundant role of StPAL in potato development. Meanwhile, StPAL6 and StPAL9 were highly active in jasmonic acid (Figure 4b and Figure 7). Studies showed that StPAL6 had one tandem replication gene, StPAL5, and StPAL9 had three tandem replication genes, StPAL10, StPAL11, and StPAL12 (Figure 3a). These four duplicated genes are most likely redundant, expressed at specific times. According to the qRT-PCR results, StPAL12 had a very high expression level at high temperatures (38 °C) and drought, so we speculated that StPAL12 could replace StPAL9 expression at high temperatures. The redundancy of the PAL gene family indicates that the PAL enzyme plays a crucial role in plant growth and development and environmental stress. Our current studies have identified many cis-acting components associated with growth and development, hormones, and anxiety (Figure 4b), such as CGTCA-Motif, ABRE, LTR, MBS, I-box, and circadian. The existence of these cis-acting elements all confirmed the regulation of StPAL expression under various stresses, such as hormone, temperature, drought, and light. As different StPAL genes contained different types, numbers, and positions of elements, this was consistent with the result of the heat map analysis of the expression profile (Figure 7b). The regions that produced these significant differences may have arisen after tandem gene duplication and recombination. As Reams and Neidle (2004) [45] reviewed, this is a beneficial mutation that enables the repeated candidate gene to be better expressed in the face of a new environment. This is also why StPALs genes overlap, but their expression patterns are different. Although the expression profiles of StPALs in potatoes are roughly similar, there are significant qualitative and quantitative differences in their regulation in plants (Figure 8), suggesting that a single StPAL protein may have different functions from other StPALs. This functional difference means that PALs are a multifunctional gene family. This phylogenetic difference was fully supported by gene structure (Figure 3b) and analysis of cis-acting elements (Figure 4b).

Many studies have shown that the PAL gene is highly expressed under low temperature stress. For example, all PALs of cucumber seedlings are up-regulated under low temperature pressure [33]. After cold treatment, the expression of PAL in walnut gradually increased over time and reached the highest level at 48 h [35]. There are other similar cases of leaves of tetraploid *I. indigotica* under cold stress [46]. *Arabidopsis thaliana* mainly regulates and enhances plant resistance to freezing by activating BR signaling through enzymes [47]. However, the signaling mechanism of potato for freezing and high temperature needs to be further studied. At present, there has been no experiment on PAL with high temperature treatment, so it is speculated that there is no element directly corresponding to the high temperature reaction detected in its cis-acting components. In this experiment, expression of STPALs was significantly up-regulated in 65% of potatoes after high temperature treatment. Combining our experimental results with previous research results, it can be found that the expression of some PAL genes will be significantly up-regulated under low temperature or high temperature stress. The results showed that the protective barrier formed by phenylpropanoid metabolism could resist cold and heat injury [48]. Indeed, PAL plays a vital role in abiotic stress as a bridge between primary metabolism and natural product synthesis. Studies have shown that, compared with drought-sensitive genotypes, the expression levels of PAL genes in the wheat root of drought-resistant genotypes are higher. Still, the expression levels of five PAL genes are deficient [49]. After drought stress, 70% of CsPALs in cucumber seedlings showed a trend of increasing and decreasing, but the overall expression level was up-regulated [33]. Under waterlogging conditions, 70% of alfalfa PAL gene expression was up-regulated [16], while wheat PAL6 expression was inhibited [50]. We found that the expression levels of only 6 of the 14 StPAL genes were significantly increased, and the expression levels of 4 genes were deficient in potato roots after drought stress treatment. Experimental results of drought stress in potato were consistent with the above experimental results of waterlogging stress, and only some genes were up-regulated. The drought regulation mechanism of *Arabidopsis* is mainly through the regulation of transcription factors by BR, which gives plants a more robust tolerance to drought stress [47]. Compared with high temperature and MeJA treatment, drought treatment only induced increased expression of StPAL8, while other StPALs did not change significantly. This may be because StPAL8 is highly involved in potato root development and regulates its lignification level [51].

We found that many kinds of cis-elements exist in potatoes, including but not limited to abscisic acid (ABRE), methyl jasmonate (CGTCA-Motif), and salicylic acid (TCA-element), etc. After ABA treatment, 85% of cucumber PAL transcripts increased [17], while walnut PAL showed no significant difference [35]. The expression of PALs in all cucumbers increased after SA treatment [33], and the presentation of PAL in walnut rose gradually in the first 24h and then decreased slightly [35]. The same situation occurred under the SA treatment in tobacco [52] and cilantro [53]. Methyl jasmonate and salicylic acid both belong to the hormones that can activate defense genes in plants and induce chemical defense in plants. In this experiment, the expression of potato PALs was induced by methyl jasmonate, and the manifestation of 60% StPALs was significantly up-regulated. The expression levels of StAPL1, StAPL2, and StAPL6 were significantly up-regulated. These results suggest that these three genes may be involved in MEJA activating plant defense mechanisms [54]. Almost all StPALs respond to defensive and abiotic stresses in response to MeJa. This is similar to the defense and response behavior of the GhPAOs gene family to low temperature. The conserved structures of individual StPAL genes may be located in intron regions, resulting in a non-highly conserved situation similar to GhPAOs [55]. Experimental results have confirmed that various stresses and plant hormones can regulate the expression of most PAL genes, and there is a significant difference in the regulation. The simultaneous presentation of multiple PAL genes explains that the role of StPAL in environmental stimulus-response is overlapped. The degree of law varies with different stresses, plants, and genes, indicating that the response of StPAL genes to high temperature, drought, and MEJA was different. This similar expression difference also exists in the GmLACS gene, and the GmLACS studied by Asma Ayaz et al. (2021) also showed different expression patterns under other stress treatments [42]. This gene expression difference is also advantageous. The introduction of various StPALs may give plants varying stress tolerance levels. The predecessors have successfully introduced a variety of tobacco penetration genes to endow crops with resistance to multiple levels of stress and improve the critical agronomic traits of crops [56].

## 4. Materials and Methods

### 4.1. Plant Materials Preparation

In this study, potato variety Desiree was used as experimental material. The experiment was carried out at the State Key Laboratory of Crop Stress Biology in Arid Areas, Northwest A&F University (107°590′–108°080′ east longitude, 34°140′–34°200′ north latitude). Tissue culture seedlings were grown on a Murashige and Skoog (MS) medium at pH 5.9 (Yang et al., 2020) containing 2% sucrose and 0.05% MES (2-morpholine-ethanesulfonic acid). The study period was from January 2022 to February 2022. The MS liquid medium containing tissue culture seedlings were grown for three weeks in an incubator at 22 °C, 16 hours light (10,000 Lx), 8 hours dark, and 70% relative humidity, and then the following treatments were performed: the tissue culture seedlings were transferred to a nutrient solution containing 10 µM methyl jasmonate, a high temperature culture environment at 38 °C, and a nutrient solution containing 20% polyethylene glycol (PEG4000) for 3 hours, 6 hours, and 24 hours, respectively. The control group was untreated seedlings. The treatment and control groups collected potato plant roots for RNA extraction. Three biological replicates were established for each treatment condition to reduce the error rate [57].

### 4.2. Identification of PAL Gene Family in Potato

Using the protein sequences of the PAL gene family identified in *Arabidopsis* thaliana (https://www.arabidopsis.org/index.jsp (accessed on 15 November 2021)) genome database as query sequences, the potato genome database was searched using local BlastP (http://solanaceae.plantbiology.msu.edu/blast.shtml (accessed on 16 November 2021)). The sequence information of the potato homologous StPAL gene family members was obtained (Table A2). The PAL gene family domain (PF00221) model file was downloaded from the Pfam database (http://pfam.xfam.org/ (accessed on 6 December 2021)) and potato protein sequences containing the PAL domain were screened using HMMER software. Then, to test whether the initial identification results were correct, the PAL gene family domain (PF00221) model file was put into SPDE2.0 software for re-screening to confirm the correctness of the results. Using the Expasy website (https://web.expasy.org/protparam/ (accessed on 7 December 2021)) and combined with the potato genome database information, we predicted and analyzed the physical and chemical properties of all PAL potato protein sequences.

### 4.3. Multiple Sequence Alignment and Phylogenetic Tree Construction

The PAL protein sequences of potatoes and *Arabidopsis* were subjected to multiple sequence alignment using Jalview to identify the various memorable domains and conserved residues. Using MEGA 11 software, the ClustalW algorithm was used to compare the potato PAL family with *Arabidopsis*, corn, rice, tobacco, grape, cassava, yam, and pine [13,14,58,59], and then a phylogenetic tree was constructed. Additionally, the statistical parameters used to build a neighbor-joining (NJ) tree were as follows: 1000 bootstrap replications and the Poisson model [60].

### 4.4. Chromosomal Location Analysis and Tandem Replicated Genes and Gene Structure

Gene location information was downloaded from the potato genome database (http://solanaceae.plantbiology.msu.edu/ (accessed on 16 November 2021)) gff3 files. The chromosome distribution of the potato PAL gene was analyzed and mapped using SPDEv2.0 software. The genes whose sequence similarity is more than 70%, that have agene interval within five genes, and a distance of less than 100 kb are defined as the tandem replicated genes [61]. The storage file of the potato PAL gene exon and intron distribution was downloaded from the potato genome database website, and we used TBtools software to draw the gene structure map.

### 4.5. Conserved Motif Identification and Cis-Acting Elements Analysis

Using the MEME website (http://meme-suite.org/ (accessed on 4 December 2021)) to analyze the conserved motifs of the potato PAL protein, we determined that the number of motif inductions was eight [62]. To study the cis-acting elements in the promoter region of the potato PAL gene, we retrieved the sequence of 2001 bp before the start codon of the PAL gene from the potato genome database and submitted it to the PlantCARE website (http://bioinformatics.psb.ugent.be/webtools/plantcare/html/ (accessed on 21 December 2021)) to predict cis-acting elements [63]. We then used SPDE2.0 software to plot the distribution of cis-acting components in the promoter region.

### 4.6. Analysis of PAL Protein Interaction in Potato

We uploaded 14 StPAL proteins of potato to the STRING website (http://cn.string-db.org/ (accessed on 28 December 2021)), selected *Arabidopsis thaliana* as the model plant, and confirmed that the 14 protein sequences corresponded to proteins in *Arabidopsis*. After the results were derived, the frequency of protein nodes was calculated using Microsoft Excel, and the protein interaction network diagram was drawn using Cytoscape software.

### 4.7. Interspecific Collinearity Analysis of PAL Gene in Potato

We downloaded the DNA files and gff3 files of the two plants from the potato genome database (http://solanaceae.plantbiology.msu.edu/ (accessed on 15 November 2021)) and the *Arabidopsis* thaliana genome database (https://www.arabidopsis.org/ (accessed on 15 November 2021)), respectively. We used the TBtools software to map the collinearity of potato and *Arabidopsis* genes, and the collinearity of PAL genes was labeled.

### 4.8. Tissue Expression and Stress Treatment Expression Analysis of the Potato PAL Genes

According to the potato transcriptome sequencing data downloaded from the PGSC website (http://solanaceae.plantbiology.msu.edu/dm_v6_1_download.shtml/ (accessed on 16 November 2021)), we deleted all the PAL genes with an FPKM value of less than 1 under tissue and stress, then calculated the Log2 value, and used SPDE 2.0 software to make a heat map [64].

### 4.9. RNAIsolation and qRT-PCR Analysis

The total RNA from potato was extracted using the TRIGene Total RNA Extraction Reagent (GenStar, Shenzhen, China, P118-01), and then Evo M-MLV RT Kit with gDNA Clean for qPCR II (Accurate Biology, Changsha, China, AG11711) was used to perform reverse transcription, according to the manufacturer’s instructions. The design of StPAL gene-specific primers for quantitative real-time PCR (qRT-PCR) analysis was investigated using the Primer Premier 6 software and NCBI. The ef1α gene was used to normalize the results (Table A4). The qRT-PCR process was performed on the Q7 Real-Time PCR System. In qRT-PCR experiments, the following thermal cycling conditions were applied: initial activation of 94 °C for 2 min, then 40 cycles of 94 °C for 15 s, 60 °C for 15 s, and 72 °C for 30 s. The relative expression levels were calculated using the comparative 2^−∆∆CT^ method [65].

## 5. Conclusions

This study identified 14 StPAL genes from the potato for the first time, distributed on 4 chromosomes, 13 of which had MIO domains. Although StPAL3 does not contain the MIO domain, qRT-PCR results suggest that it is still involved in the stress response under high temperature and MeJa stress. Phylogenetic tree analysis showed that 12 StPALs were closely related to the tobacco PAL gene family. Analysis of cis-acting elements revealed that most StPALs are involved in defense responses to abiotic stresses, such as hormones and adverse environments. The qRT-PCR study showed that StPAL1, StPAL6, StPAL8, StPAL12, and StPAL13 were involved in the response mechanism of potato to high temperature and drought stress, while MeJa could significantly up-regulate the expression of StPAL1, StPAL2, and StPAL6, indicating that these genes were involved in potato chemical defense mechanism. These three stresses significantly inhibited the expression of StPAL7, StPAL10, and StPAL11, once again proving that PAL is a multifunctional gene family, which may give plants resistance to multiple and different stresses. Genome-wide identification of the potato PAL gene family will allow us to gain a more comprehensive understanding of the diversity of this family. In addition, although the protein interaction network map revealed the potential function of the StPAL protein, it still needs to be determined with more in-depth research and analysis. This study aims to provide valuable insights for the subsequent functional validation of these genes.

## Figures and Tables

**Table 1:** The PAL genes in potato and properties of the deduced proteins (*Solanum tuberosum*).

Gene ^1^	Gene ID ^1^	Chromosome Location ^1^ (bp) ^1^	ORF Length (bp) ^1^	No. of Exons ^1^	Protein ^2^			Subcellular Localization
					Length (aa)	MW (Da)	pI	
StPAL1	Soltu.DM.03G004870	Chr03:5254948—5259172 (+)	2171	2	723	78,585.86	6.11	Cytoplasm
StPAL2	Soltu.DM.03G004900	Chr03:5293236—5296444 (+)	2172	2	723	78,749.96	6.00	Cytoplasm
StPAL3	Soltu.DM.03G004920	Chr03: 5304263—5308005 (+)	2172	5	708	77,814.62	6.23	Plasma membrane
StPAL4	Soltu.DM.03G011440	Chr03: 31804241—31801966 (−)	1956	4	651	70,751.77	6.75	Cytoplasm
StPAL5	Soltu.DM.03G011480	Chr03: 31877150—31874688 (−)	2022	2	673	73,124.54	6.21	Cytoplasm
StPAL6	Soltu.DM.03G011490	Chr03: 31902002—31895849 (−)	2142	3	713	77,929.94	7.97	Plasma membrane
StPAL7	Soltu.DM.05G017030	Chr03: 40539165—40534755 (−)	2004	2	667	73,689.12	5.41	Cytoplasm
StPAL8	Soltu.DM.05G026870	Chr05: 54865269—54869381 (+)	2124	2	707	77,510.48	6.07	Cytoplasm
StPAL9	Soltu.DM.09G005690	Chr09: 5255916—5252152 (−)	2169	2	722	78,590.86	6.32	Cytoplasm
StPAL10	Soltu.DM.09G005700	Chr09: 5262994—5260062 (−)	2169	2	722	78,488.71	6.04	Cytoplasm
StPAL11	Soltu.DM.09G005710	Chr09: 5280698—5283618 (+)	2160	2	719	78,330.48	6.15	Cytoplasm
StPAL12	Soltu.DM.09G005720	Chr09: 5287714—5290290 (+)	2172	2	723	78,819.07	6.00	Cytoplasm
StPAL13	Soltu.DM.10G005900	Chr10: 5983790—5987501 (+)	2130	2	709	77,707.78	6.16	Cytoplasm
StPAL14	Soltu.DM.10G020990	Chr10: 52868738—52864254 (−)	2136	2	711	77,543.40	5.86	Cytoplasm

^1^ Gene information was retrieved from the *S. tuberosum* v6.1 genome annotation (http://solanaceae.plantbiology.msu.edu/dm_v6_1_download.shtml (accessed on 21 November 2020)). ^2^ Protein profile information from the ExPASy-ProtParam tool (https://web.expasy.org/protparam/ (accessed on 21 November 2020)).

**Table 2:** List of the conserved motifs of StPAL proteins.

Motif	Length	Amino Acid Sequence
Motif1	50	QKPKQDRYALRTSPQWLGPQIEVIRAATKMIEREINSVNDNPLIDVSRNK
Motif2	50	DYGFKGAEIAMASYCSELQFLANPVTNHVQSAEQHNQDVNSLGLISARKT
Motif3	50	SDWVMDSMSKGTDSYGVTTGFGATSHRRTKNGGALQKELIRFLNAGVFGN
Motif4	50	VMNGKPEFTDYLTHKLKHHPGQIEAAAIMEHILDGSSYVKAAQKLHEMDP
Motif5	50	HTLPHSATRAAMLVRINTLLQGYSGIRFEILEAITKLINSNITPCLPLRG
Motif6	50	ELHPARFCEKELLRVVDREYLFAYADDPCSSNYPLMQKLRQVLVDHAMKN
Motif7	50	NRITECRSYPLYRLVRZELGTELLTGEKVRSPGEEIDKVFTAMCNGQIID
Motif8	50	VSGGFFELQPKEGLALVNGTAVGSGMASIVLFESNILAVMSEVLSAIFAE

## Data Availability

Not applicable.

## Appendix A

**Table A1:** Protein sequences as used in the phylogenetic relationship analysis.

Species	Gene Name	Locus ID
*Arabidopsis thaliana*	AtPAL1	AT2G37040
	AtPAL2	AT3G53260
	AtPAL3	AT5G04230
	AtPAL4	AT3G10340
*Dioscorea cayenensis*	DcPAL1	XP_039137778
	DcPAL2	XP_039132878
*Manihot esculenta Crantz*	MePAL1	XP_021620397
	MePAL2	XP_021611089
	MePAL3	AAK62030
	MePAL4	XP_021597003
	MePAL5	XP_021618700
*Nicotiana tabacum*	NtPAL1	M84466
	NtPAL2	D17467
	NtPAL3	X78269
	NtPAL4	EU883669/70
*Oryza sativa* L.	OsPAL1	XP_015620761
	OsPAL2	XP_015640196
	OsPAL3	NP_001388835
	OsPAL4	XP_015625125
	OsPAL5	XP_015625126
	OsPAL6	XP_015626729
	OsPAL7	BAG94561.1
	OsPAL8	XP_015633749
	OsPAL9	XP_015615944
*Pinus pinaster*	PpPAL1	AAT66434
*Solanum tuberosum*	StPAL1	Soltu.DM.03G004870
	StPAL2	Soltu.DM.03G004900
	StPAL3	Soltu.DM.03G004920
	StPAL4	Soltu.DM.03G011440
	StPAL5	Soltu.DM.03G011480
	StPAL6	Soltu.DM.03G011490
	StPAL7	Soltu.DM.05G017030
	StPAL8	Soltu.DM.05G026870
	StPAL9	Soltu.DM.09G005690
	StPAL10	Soltu.DM.09G005700
	StPAL11	Soltu.DM.09G005710
	StPAL12	Soltu.DM.09G005720
	StPAL13	Soltu.DM.10G005900
	StPAL14	Soltu.DM.10G020990
*Vitis vinifera* L.	VvPAL1	XP_002267953
	VvPAL2	XP_003633985
	VvPAL3	XP_002268181
	VvPAL4	NP_001384847
	VvPAL5	XP_003633986
	VvPAL6	XP_002268732
	VvPAL7	RVW78684
	VvPAL8	XP_010662075
	VvPAL9	XP_002285277
	VvPAL10	RVW84295
	VvPAL11	XP_002281799
	VvPAL12	RVW78687
*Zea mays* L.	ZmPAL1	NP_001147433
	ZmPAL2	NP_001151482
	ZmPAL3	NP_001147922
	ZmPAL4	NP_001105334
	ZmPAL5	NP_001141469
	ZmPAL6	XP_008645952
	ZmPAL7	NP_001168086
	ZmPAL8	XP_020402425

**Table A2:** The FPKM values of phenylalanine ammonia-lyase genes in various tissues.

Gene	Leaves	Roots	Shoots	Callus	Stolons	Tubers	Flowers	Petioles	Petals	Stamens	Carpels
StPAL1	0.00	0.41	2.34	1.88	7.21	4.99	0.00	0.48	0.00	0.00	0.00
StPAL2	0.00	1.07	2.96	2.51	5.96	4.78	0.00	0.00	0.00	0.00	0.00
StPAL3	0.00	0.00	0.00	0.00	6.17	4.18	0.00	0.00	0.00	0.00	0.00
StPAL4	0.00	0.00	3.15	0.67	8.19	4.28	0.00	1.85	3.10	0.00	0.00
StPAL5	0.79	1.29	5.94	3.30	9.11	7.25	2.04	1.99	0.00	0.00	0.00
StPAL6	0.17	0.00	3.67	0.00	7.58	4.40	0.00	2.27	3.47	0.00	0.62
StPAL7	0.00	0.00	0.00	0.20	0.00	0.00	0.00	0.00	0.00	0.00	1.49
StPAL8	1.67	3.81	3.17	5.98	5.04	4.12	3.46	3.22	3.60	4.39	3.84
StPAL9	6.84	7.89	7.47	9.48	9.79	8.29	7.82	5.74	9.33	3.34	7.77
StPAL10	3.88	1.85	4.63	3.91	7.52	6.33	5.57	3.42	3.04	0.00	4.07
StPAL11	0.64	0.45	3.63	2.30	7.40	7.07	0.23	3.23	0.00	0.00	1.90
StPAL12	0.00	0.00	0.00	0.87	0.00	0.00	0.00	0.11	0.05	0.00	0.00
StPAL13	2.31	2.40	4.48	7.71	3.10	1.32	2.36	3.93	3.56	0.36	0.92
StPAL14	5.25	4.24	4.66	5.63	5.86	5.71	7.45	5.34	4.83	9.12	5.59

**Table A3:** The FPKM values of phenylalanine ammonia-lyase gene in various treatments.

Gene	Salt	Mannitol	Heat	*P. infestans*	BABA	BTH	ABA	IAA	GA3	BAP
StPAL1	−0.31	−1.01	−0.55	0.03	−3.13	0.07	0.35	0.85	0.99	1.27
StPAL2	0.79	−0.73	−0.89	−1.12	−3.23	−0.72	1.37	1.68	1.91	0.05
StPAL3	0.00	2.53	−0.34	1.24	−0.27	−0.49	−0.88	−1.09	−0.64	0.00
StPAL4	−1.83	−2.13	1.55	1.12	−1.37	−2.94	−1.52	−1.17	−2.94	0.00
StPAL5	1.49	2.01	0.87	−0.23	−0.38	−0.66	0.98	1.11	1.83	1.23
StPAL6	−1.76	1.63	0.65	−0.02	−2.69	2.14	2.94	4.09	2.35	0.00
StPAL7	0.00	0.00	−1.04	−0.42	−1.41	0.00	0.00	0.00	0.00	0.00
StPAL8	−0.42	−0.86	−0.50	1.65	−1.22	0.35	−0.12	0.74	0.10	1.29
StPAL9	0.20	−0.48	0.25	0.97	−1.12	0.21	0.20	0.52	−0.13	0.52
StPAL10	−0.32	−0.54	−0.63	0.16	−2.27	0.27	0.23	0.38	−0.25	1.40
StPAL11	0.32	0.15	−0.98	−0.45	−2.12	−0.57	1.08	0.70	0.99	1.02
StPAL12	0.00	0.29	0.74	−0.98	−0.98	0.00	0.11	0.00	0.18	0.00
StPAL13	0.24	−0.34	−1.57	0.12	−1.57	0.30	−0.10	0.26	−0.45	−1.76
StPAL14	0.04	−0.33	−1.01	−0.47	0.51	0.46	−0.26	0.37	−0.98	1.28

**Table A4:** The primer sequences used in qRT-PCR analyses.

Species	Sequence (in 5′ → 3′ Order)	
	Forward	Reverse
StPAL1	TCTAATCTGACAGCAGGAAGGA	CCGAGCAGTAAGAAGCCATC
StPAL2	CCTCGGGTGATCTTGTACCTT	TAACACCAGCCACACGGAAA
StPAL3	GAATGGCACAGCAGTTGGT	TTCCGTTCATCACTTCAGCAA
StPAL4	TGCACAAAATGGACATAAAGCCAA	AGAAAGTTCCACTTTGACCCCAC
StPAL5	ATTACCCCGTGTTTGCCCCT	ACCATTGGGTCCAACAGCCT
StPAL6	GCCATCTAATCTCACAGCAGGAAGG	AAGTTCCGAGCAGTAAGAAGCCATC
StPAL7	GCCGGTGATCCGACTAGGTG	CCGACAGCTCCACCTTCACA
StPAL8	TGCAGCCCTACAGAAAGAGC	CCTCACTAGCATAGCTGCCC
StPAL9	TGCTGATGATCCCTGCAGTTCA	GGGTTGCCACTTTCAAGCATAG
StPAL10	TCTTGAAAGTGGCAACCCTGT	CTCCTCACCGGGAGATCGAA
StPAL11	GCTATGCTTGAGAGTGGAAACCC	GTCAATCTCCTCACCGGGCG
StPAL12	CCCGTTGTCATACATTGTTGGG	GCTGTGCCATTCACAAGTGC
StPAL13	TGCTGAAGAAGCGTTCCGTGTT	ATGCCATACCAGAGCCAACTGC
StPAL14	GCCAGAGTCGCGTTGGAAAG	CAATTCCGTCCCGAGCTCCT
ef1α	GGAAAAGCTTGCCTATGTGG	CTGCTCCTGGCAGTTTCAA
